# Flexible liposomal gel dual-loaded with all-trans retinoic acid and betamethasone for enhanced therapeutic efficiency of psoriasis

**DOI:** 10.1186/s12951-020-00635-0

**Published:** 2020-05-24

**Authors:** Wei Wang, Gao-feng Shu, Kong-jun Lu, Xiao-ling Xu, Min-cheng Sun, Jing Qi, Qiao-ling Huang, Wei-qiang Tan, Yong-zhong Du

**Affiliations:** 1 Department of Pharmacy, The Third People’s Hospital of Hangzhou, 38 West Lake Avenue, Hangzhou, 310009 China; 2 Institute of Pharmaceutics, College of Pharmaceutical Sciences, Zhejiang University, 866 Yuhangtang Road, Hangzhou, 310058 China; 3 Department of Plastic Surgery, Sir Run Run Shaw Hospital, Zhejiang University School of Medicine, 3 East Qingchun Road, Hangzhou, 310016 China

**Keywords:** Flexible liposomes, All trans-retinoic acid, Betamethasone, Enhanced anti-psoriatic efficacy

## Abstract

**Background:**

Psoriasis is a chronic immune-mediated inflammatory skin disease without effective treatment. The utilization of all trans-retinoic acid (TRA) and betamethasone (BT) for the treatment of psoriasis is still facing difficulties, due to their relatively poor stability, limited skin permeation, and systemic side effects. Flexible liposomes are excellent in deeper skin permeation and reducing the side effects of drugs, which is promising for effective treatment of skin disorders. This work aimed to establish dual-loaded flexible liposomal gel for enhanced therapeutic efficiency of psoriasis based on TRA and BT.

**Results:**

Flexible liposomes co-loaded with TRA and BT were successfully prepared in our study. The characterization examination revealed that flexible liposomes featured nano-sized particles (around 70 nm), high drug encapsulation efficiency (> 98%) and sustained drug release behaviors. Flexible liposomes remarkably increased the drug skin permeation and retention as compared with free drugs. Results on HaCaT cells suggested that flexible liposomes were nontoxic, and its cellular uptake has a time-dependent manner. In vivo studies suggested the topical application of TRA and BT dual-loaded liposomal gel had the best ability to reduce the thickness of epidermal and the level of cytokines (TNF-α and IL-6), largely alleviating the symptoms of psoriasis.

**Conclusions:**

Flexible liposomal gel dual-loaded with TRA and BT exerted a synergistic effect, which is a promising topical therapeutic for the treatment of psoriasis.
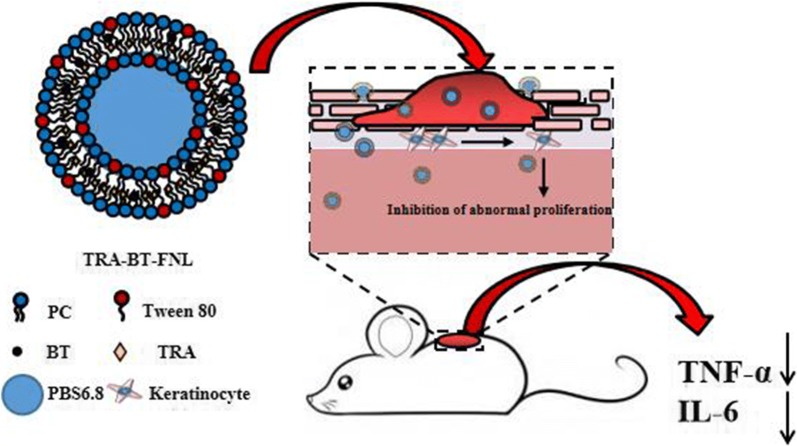

## Introduction

Psoriasis is considered to be the most prevalent chronic immune-mediated inflammatory skin disease [1]. This disease is commonly expressed as the long-lasting presence of red and sharp scaly plaques on the skin, seriously reducing the patient’s quality of life. Previous studies have reported that psoriasis often lead to increased risks of comorbidities, such as inflammatory arthritis, obesity, hypertension, and inflammatory bowel disease [2]. Unfortunately, the incidence of psoriasis is increasing year by year, with approximately 2–4% of population suffering from this disease in the world [3]. Current treatments of psoriasis mainly include topical therapy [4], phototherapy [5], immunotherapy [6], and systemic therapy [7]. Among them, topical therapy is the most frequently used mean for the treatment of psoriasis, due to its convenience and reduced side effects [8]. However, the topical administration of conventional formulations is still not satisfactory, which is associated with the limited drug penetration and decreased patient compliance [9]. Thus, exploring a novel topical treatment with enhanced therapeutic effect is largely needed for patients who have psoriasis.

All-trans retinoic acid (TRA), a member belongs to retinoid family, is frequently used for the treatment of skin disorders, such as acne, skin carcinoma, wound recovery, and psoriasis [10–12]. The potential of TRA in psoriatic treatment is partly due to the fact that TRA can regulate the skin cell growth and differentiation, sebum synthesis, and collagen production by binding with the retinoic acid receptors [13, 14]. Previous study has suggested that TRA could inhibit the inflammatory response by largely reducing the expression levels of proinflammatory cytokines (e.g., IL-6, ICAM-1 and HLA-DR), which is also beneficial for psoriatic treatment [15–17]. Although TRA is a FDA-approved drug for different skin diseases, its wide utilization in clinic is still largely restrained by its poor stability against oxygen and light, water insolubility, and skin irritation [18]. Thus, it is highly needed to design new approaches for overcoming these difficulties mentioned above.

Betamethasone (BT), as a type of glucocorticoids, is also effective in term of alleviating the symptoms of psoriasis [19]. BT has a number of functions, including anti-inflammatory, vasoconstrictive, apoptotic, antimitotic and immunomodulatory abilities [20, 21]. It is well established that these functions are closely related to the ability of BT to treat psoriasis [20, 22]. However, a major problem related to the topical application of BT is its long-term usage can induce adverse skin reactions, such as local atrophy and pigmentation [23].

Combination therapy is considered to be one of the most promising ways to overcome the compensatory mechanisms, as well as to reduce the dose-related adverse off-target effects. The combinational use of two or more drugs with different therapeutic mechanisms has advantages of achieving an enhanced the therapeutic effect via a multi-target therapeutic approach [24]. Glucocorticoids have strong anti-inflammatory and anti-proliferative effects, which could inhibit the prostaglandins or cytokines in epidermal cells, thereby reducing the skin response of TRA which could reduce the stimulation of TRA [25]. TRA can prevents glucocorticoid-induced changes in the connective tissue matrix, maintain the collagen, glycosaminoglycan and fibronectin at normal levels, and therefore reverse the skin atrophy caused by glucocorticoid treatment [26]. Previous studies has pointed out that the topical administration of ointments containing both TRA and BT results in a synergistic efficacy and reduced side effect during psoriatic treatment [27, 28]. However, the formulations in their works were prepared by directly mixing BT and TRA within the ointments, therefore having a relatively poor skin penetration.

Liposome-based carriers are nontoxic and biodegradable, which are good candidates to co-deliver multiple drugs to the action regions. Liposome-based drug delivery systems also have excellent skin penetration and drug protection, which is promising for effective treatment of skin disorders [29]. Emerging as the new generation of liposomes, flexible liposomes composed of phospholipids and an edge activator (e.g., Tween 80 and sodium cholate) has attracted considerable attentions in recent years [30]. The presence of edge activators provides the flexible liposomes with high flexible membranes, which could result in an enhanced skin penetration than the conventional ones with rigid membranes [31, 32]. To our best knowledge, studies associated with the utilization of flexible liposomes containing TRA and BT for synergistic treatment of skin disorders such as psoriasis have not been reported by now.

Topical gels are commonly prescribed for use in patients with inflammatory-related skin diseases, such as psoriasis. The utilization of topical gels is convenient, but also it could provide a prolonged retention capacity of the drug on the skin, which is beneficial for the treatment of skin disease [33, 34]. Carbomers are acid-based polymers that are commonly used to fabricate gels for skin care and skin disease treatment. The utilization of carbomer-based gel has a number of advantages [35, 36]: (i) it can provide a high viscosity at a relatively low concentration; (ii) it has excellent bioadhesive and viscoelastic properties; (iii) it has extremely low toxicity and irritant potential, which is widely accepted by many patients with skin disease.

Thus, the objective of our study was to prepare flexible liposomes co-loaded with TRA and BT for the synergistic treatment of psoriasis. The physicochemical parameters (such as average particle size, size distribution, morphology observation, and in vitro drug release) of the flexible liposomes were systematically characterized, followed by the investigation of in vitro cytotoxicity and cellular uptake using HaCaT cells. Then, a flexible liposome-based gel was prepared for topical drug delivery, as well as the convenience for use. Finally, in vivo anti-psoriatic activities of different liposomal gel were evaluated by using psoriatic mice model induced by IQM.

## Materials and methods

### Materials

Lecithin was purchased from Lipoid GmbH (Ludwigshafen, Germany). All-trans retinoic acid (TRA), fluorescein isothiocyanate (FITC) were purchased from SinopharmDalian Meilun Biotechnology Co., Ltd (Dalian, China). Betamethasone was purchased from Tianjin Tianyao Pharmaceutical Co., Ltd (Tianjing, China). Fetal bovine serum (FBS) was purchased from Hangzhou Tianhang Biotechnology Co., Ltd. (Hangzhou, China). Dulbecco’s minimum essential medium (DMEM), Trypsin-0.02% EDTA was purchased from Geno Biomedical Technology Co., Ltd. (Zhejiang, China). 5% Imiquimod cream was purchased from Hubei Keyi Pharmaceutical Co., Ltd (Hubei, China). ELISA kits (Mouse TNF-α, IL-6) were purchased from Jiangsu Meimian Industrial co., Ltd (Jiangsu, China). Primary antibody (CD3), secondary antibody Hrp-goat anti-rabbit, hematoxylin dye, and eosin stain were purchased from Servicebio co., Ltd (Hubei, China). Bull Serum Albumin (BSA) was purchased from Solarbio co., Ltd (Beijing, China). Other reagents or solvents were chromatographic or analytical pure grade.

### Cell culture and animals

Immortalized human keratinocytes (HaCaT) was obtained from American Type Culture Collection (ATCC, Rockville, USA). HaCaT cells were cultured in DMEM supplemented with 10% of fetal bovine serum in a humidified atmosphere (37 °C, 5% CO_2_). BALB/c mice (18–22 g) were purchased from Zhao Yan New Drug Research Center co., Ltd (Suzhou, China). All mice were pathogen-free and had free access to water and food. All animal experiments were conducted in accordance with the guidelines formulated by the animal care and use committee of Zhejiang University.

### Preparation of flexible nanoliposomes

Flexible nanoliposomes co-loaded with TRA and BT (TRA-BT-FNL) were prepared by a thin-film hydration method. Briefly, 400 mg of lecithin, 160 mg of Tween-80, 10 mg of TRA, and 10 mg of BT were dissolved in 10 mL of absolute ethanol. The ethanol was then removed by evaporation at 40 °C under dark condition. The formed membrane was then hydrated with 5 mL of PBS (pH = 6.8) for an appropriate time in an ultrasonic water bath. Finally, the resultant mixture was ultra-sonicated for 4 min by a probe sonicate to obtain the well-dispersed flexible liposome suspension. The control groups of flexible nanoliposomes loaded with a single drug or without drug were also prepared by the same method.

### Preparation of liposomal gel

The blank gel was prepared by dissolving 1 wt% of carbomer 940, 10 wt% of glycerin, 0.1 wt% of EDTA and 0.1 wt% of ethylparaben in distilled water, followed by adjusting the pH to 6.5 using triethanolamine. The blank gel was then mixed with flexible liposome suspensions at a ratio of 75:25 (w/w) to formulate the liposomal gel. The corresponding contents of TRA and BT in the liposomal gel were 0.05 wt%. The gel containing the same amount of free TRA or BT were also prepared by the same method.

### Characterization of flexible nanoliposomes

The average particle size and size distribution of flexible liposomes were examined by a dynamic light scattering (DLS) instrument (litesizer 500, Anton-Paar, Austria) after suitable dilution. Morphology measurements of flexible liposomes were performed by a transmission electron microscopy (TEM, JEM-1230, JEOL, Tokyo, Japan). The drug loading (DL) and encapsulation efficiency (EE) of flexible liposomes were carried out by the ultrafiltration centrifugation method. In brief, a certain amount of flexible liposome suspensions were ultrafiltration centrifuged (MWCO:3.5KD) at 5000 r/min for 10 min to separate the unencapsulated drugs. The concentrations of encapsulated drugs in the filtrate, as well as the total drug in the formulations were detected by high performance liquid chromatography (HPLC) method. DL and EE were then calculated according to the following Eq. 1 and Eq. 2, respectively.

1 $${\text{DL (\%)}} = \frac{\text{Total mass of drug} - \text {mass of unencapsulated drug}}{\text{Total mass of liposomes}} \times 100{\text{\% }}$$

2 $${\text{EE (\%)}} = \frac{\text{Total mass of drug} -\text{mass of unencapsulated drug}}{\text{Total mass of drug}} \times 1 0 0 {\text{\% }}$$

The chemical stability of liposomes was investigated by measuring the degrading efficiency of TRA under sunlight or oxygen conditions. TRA and BT solution (dissolved in 50% DMAO/propylene glycol) were used as controls. For the photostability study, direct sunlight of clear days of mid-April of Hangzhou City, China was used as the light source. The liposomes loaded with BT and TRA were placed in transparent glass containers and then filled with nitrogen gas. The samples were then exposed to the sunlight for a predetermined time (0–80 min). For the oxygen stability, the liposome samples were placed in direct in the air without sealing and light for 5 days. The change in TRA content during storage were measured by HPLC method. The degrading efficiency of TRA were calculated by Eq. 3.

3 $${\text{Degrading efficiency (\%)}} = \frac{\text{Total loss of TRA}}{\text{Total mass of TRA in liposomes}} \times 1 0 0 {\text{\% }}$$

### In vitro drug release

In vitro drug release behaviors of flexible nanoliposomes were analyzed by the dialysis method. Briefly, a known amount of free drug or drug-loaded flexible nanoliposomes was placed in a dialysis bag (MWCO:3.5KD). The dialysis bag was then immersed in the release medium, which contained a mixture of ethanol and PBS (30:70, v/v, pH 6.8). After constant stirring at 100 rpm (37 °C) for predefined time points, the release medium was removed and then replaced by the same volume of fresh medium. The concentrations of TRA and BT in the release medium were determined by HPLC.

### In vitro skin permeation and retention

Full thickness skin was firstly obtained from the ventral part of SD rats. After removing the extraneous subcutaneous fat, the rat skin was stored at − 80 °C prior to use. In vitro skin permeation and retention of different formulations were performed by using Franz diffusion cells. In brief, a suitable area of skin sample was fixed on the Franz diffusion cells, with the corneum and dermis layers facing the donor and receptor chambers, respectively. One gram of liposomal gel sample was placed on the surface of skin, liposomes and free drug solution were used as references. 7 mL of release medium (ethanol/PBS 30:70, pH 6.8) was filled into the receptor chamber, which was then constantly stirred at 100 rpm (37 °C). At prescribed time intervals, 1 mL of release medium was withdrawn, and immediately supplemented with 1 mL of fresh medium. The contents of TRA and BT in the release medium were analyzed by HPLC.

At 24 h post treatment, the skin was washed with ice-cold PBS for three times to remove the residual formulations. An appropriate amount of skin was minced by a tissue homogenizer. The TRA and BT in the skin tissues were extracted by methanol, and then their contents were quantified by HPLC.

### In vitro Cytotoxicity

The cytotoxicity of flexible liposomes loaded with or without drugs towards HaCaT cells was evaluated by MTT assay. In brief, HaCaT cells were seeded in 96-well plate with a density of 5 × 10^3^ cells per well, followed by incubation for 24 h prior to formulation addition. The cells were then allowed to incubate with different type of formulations at concentrations of 0–1000 μg/mL for another 48 h. After that, 20 μL of MTT solution (5 mg/mL) was added into each well, followed by incubation for another 4 h. After removing the supernatant, the purple precipitate in each wall was dissolved by adding 150 μL of DMSO under constant shake at 96 rpm (37 °C) for 30 min. The cell viability was then calculated by measuring the absorbance at 570 nm, with the untreated cells as control group.

### Cellular uptake

The cellular uptake of flexible liposomes was monitored by a fluorescence inverted microscope and flow cytometry. Octadecylamine-fluorescein isothiocyanate (ODA-FITC), which was used as a fluorescent probe, was synthesized by the method reported in a previous study [37]. ODA-FITC loaded flexible liposomes (ODA-FITC-liposome) were prepared by the aforementioned thin-film hydration method, which were then used for the following cellular uptake study. In brief, HaCaT cells were seeded in a 12-well plate at a density of 5 × 10 ^4^ cells/well, which was then allowed to adherently grow overnight. The cells were then treated with flexible liposomes with final ODA-FITC concentration of 10 μg/mL for prescribed times of 2–24 h. After stained with DAPI, the fluorescent images of cells were captured by a fluorescence inverted microscope (Axio Observer A1, Zeiss, Germany). In another study, the cells were collected, and the fluorescence intensity of FITC was detected by a flow cytometry (ACEA NovoCyteTM, ACEA Biosciences, American).

### In vivo skin distribution

In vivo skin distribution of liposomal gel was investigated by confocal laser scanning microscope (CLSM). Briefly, gel loaded with ODA-FITC-liposome or free ODA-FITC was prepared, which was then placed on the skin of Wistar rats under a dark condition. At 12 h post administration, the skin tissue was collected for CLSM observation.

### Establishment of psoriatic model

The establishment of psoriatic model were carried out by a method described in a previous study [38]. Briefly, 5% of imiquimod cream (IQM) was topically applied on the back skin (2 cm^2^) of BALB/c mice for total 7 days at a dose of 62.5 mg per day. To investigate whether the psoriatic model was successfully built or not, the pathological changes of skin tissue was investigated by H&E staining method.

### Psoriatic treatment

BALB/c mice were randomly divided into 8 group (5 mice per group): (1) Blank control groups: heathy mice without any treatment; (2) IQM groups: IQM-induced psoriatic mice without any treatment; (3) TRA-BT-FNL-Gel groups: IQM-induced psoriatic mice treated with TRA-BT-FNL-Gel; (4) TRA-FNL-Gel groups: IQM-induced psoriatic mice treated with TRA -FNL-Gel; (5) BT-FNL- Gel groups: IQM-induced psoriatic mice treated with BT-FNL-Gel; (6) Free-TRA-BT-FNL-Gel groups: IQM-induced psoriatic mice treated with Free-TRA-BT-FNL-Gel; (7) Free-TRA-Gel groups: IQM-induced psoriatic mice treated with Free-TRA -Gel; (8) Free-BT-Gel groups: IQM-induced psoriatic mice treated with Free-BT-Gel. The anti-psoriatic effects of different formulations towards the corresponding mice were started on the 3rd day, and the treatments were continued for 7 days. The gel containing TRA or BT was applied at a dose of 100 mg/day. Body weights of mice in each group were recorded during the treatment. After treatment, all mice were sacrificed, and their spleen were weighted and then captured by a digital camera. The corresponding dorsal region of skin samples were collected for thickness measurements and histopathological examinations. A part of skin samples was also stored at − 80 °C prior to the measurements of cytokines level.

### Scoring severity of skin inflammation

Psoriasis Skin Area and Severity Index (PASI) was used to score the skin inflammation, in which the thickness and erythema of skin (IQM-induced regions) were scored by the method reported previously [39]. The PASI score ranges from 0 to 4, in which 0, 1, 2, 3, and 4 denotes none, slight, moderate, marked, and very marked severity of erythema and thickening of the skin, respectively.

### Histopathological examination

Histopathological examination was carried out to investigate the pathological changes in the process of psoriatic treatment. The skin samples were stripped from the sacrificed mice of different treatment groups. The psoriatic skin samples were then stained with H&E, and then analyzed under an inverted microscope. The expression level of CD3 in psoriatic skin samples was detected by immunohistochemistry.

### Cytokines level determination

The cytokines levels (TNF-α and IL-6) in psoriatic skin samples were determined by enzyme-linked immunosorbent assay (ELISA) assay. A suitable amount of skin samples was mixed with PBS, and then the mixture was well homogenized by a tissue homogenizer. After centrifugation, the levels of TNF-α and IL-6 in the supernatant was determined by ELISA assay according to the manufacture’s introduction.

### Skin compliance study

Skin compliance study of liposomal gel was performed on the healthy skin of Wistar rats. Liposomal gels were placed on the normal skin at a drug dose of 100 mg/day for total 7 days. The severity of skin irradiation caused by different types of liposomal gel was recorded and scored during the treatment. The score criterion of skin irradiation was listed in Table 1.

**Table 1:** Grading criteria for skin irritation experiments

Erythema score	Expression	Edema scores	Expression
0	Non	0	Non
1	Slight	1	Slight
2	Obvious	2	Obvious
3	Severe	3	Severe edema (the skin bulges about 1 mm)
4	Purplish red spot with eschar	4	Severe edema (skin swelling over 1mm with enlargement)

The total score of the skin irritation test was 8, and the rating was non-irritation (0–0.49), mild irritation (0.5–2.99), moderate irritation (3.0–5.99), and severe irritation (6.0–8.0)

### Statistical analysis

All data are expressed as the mean ± standard deviation (SD) of three separate experiments. One-way analysis of variance (ANOVA) was used to determine the differences between the obtained data. p value < 0.05 was considered as statistically significant difference.

## Results

### Characterization of flexible liposomes

**Fig. 1:**
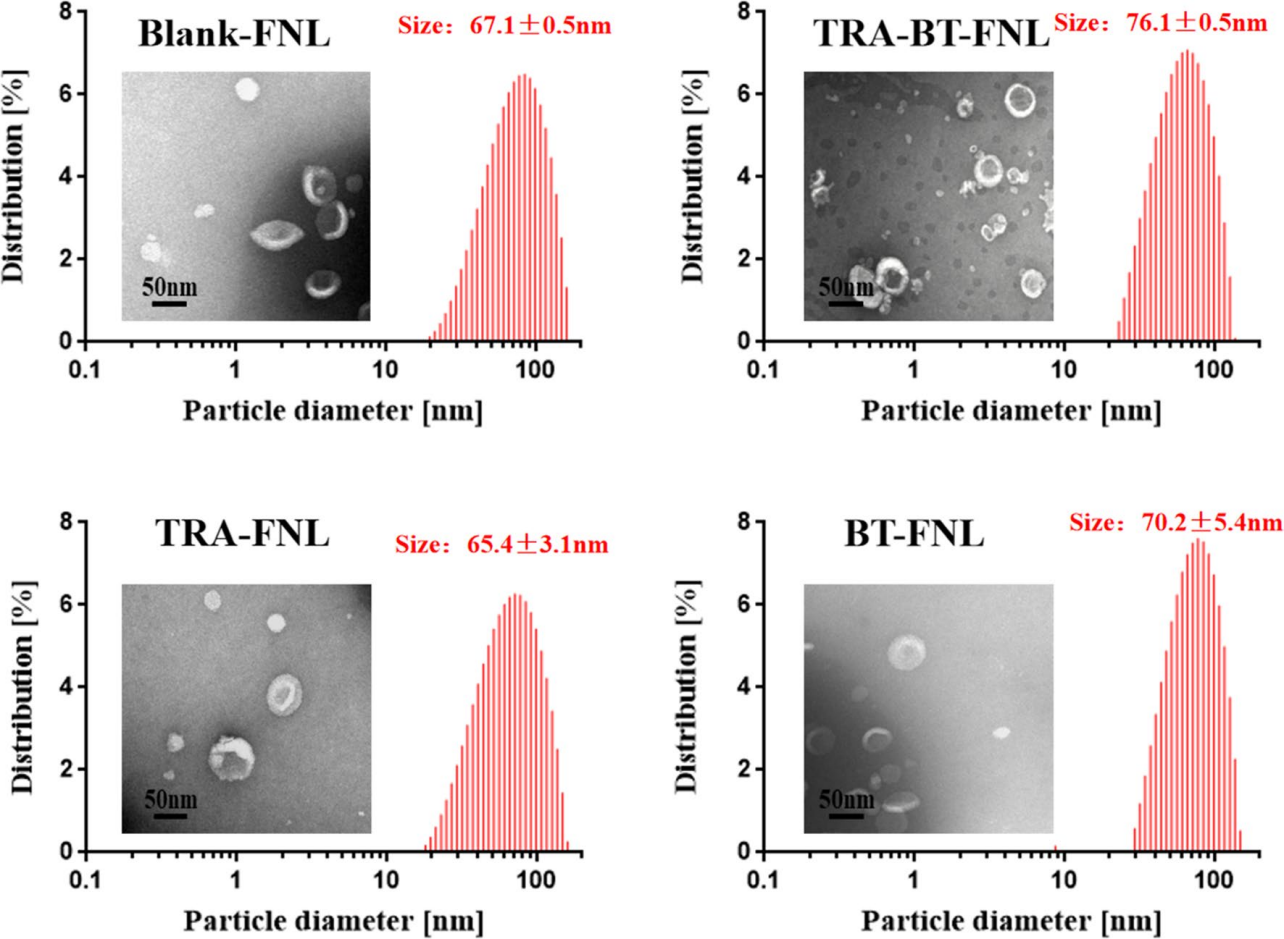
Particle size, size distribution and transmission electron micrograph of flexible nano-liposomes

The characterization of liposome-based delivery systems is fundamental, since it commonly affects the biopharmaceutical behaviors of the drugs in the delivery process. Thus, we firstly measured some physicochemical parameters of the flexible liposomes, including average particle size, polydispersity index, drug encapsulation efficiency and loading capability. DLS measurements showed that the average particle sizes of blank-FNL, TRA-BT-FNL, TRA-FNL and BT-FNL were 67.1 ± 0.5 nm, 76.1 ± 0.5 nm, 65.4 ± 3.1 nm and 70.2 ± 5.4 nm, respectively (Fig. 1). The corresponding PDI of those liposomes were 0.230 ± 0.006, 0.251 ± 0.009, 0.218 ± 0.010 and 0.235 ± 0.011, respectively. TEM images revealed that all flexible liposomes had a hollow spherical morphology, and had similar particle sizes with those of DLS results. Liposomes with small particle size of < 70 nm are considered to be promising carriers for dermal delivery, due to their excellent skin penetration and deposition [40]. Therefore, the flexible liposomes prepared in our study were highly suitable for topical application. The drug encapsulation efficiencies of all formulations were around 99%, and their drug loading capabilities were calculated to be around 2% (Table 2). The small particle size and high drug encapsulation efficiency suggested that flexible liposomes co-loaded with TRA and BT were successfully fabricated in our study. The oxygen and light stabilities of TRA in liposomes were also investigated in our study. It was found that TRA was very unstable in solution, with around 80% of TRA was loss after 80 min of exposure under sunlight conditions (Additional file 1: Figure S1a). In comparison, the degrading efficiency of TRA in liposomes were much lower, showing that less than 40% of TRA was loss at the same exposure time. The enhanced light stability of TRA in liposomes were also reported in other previous studies [11, 41]. In the case of oxygen stability, the degrading efficiency of TRA in liposomes was much slower than those in TRA in liposomes could be attributed to that liposome layers may retard oxygen diffusion, thereby leading to the reduction of TRA oxidation (Additional file 1: Figure S1b). Overall, these results suggested that the oxygen and light stabilities of TRA could been significantly improved by encapsulating them in liposomes.

**Table 2:** Encapsulation efficiency and drug loading of TRA and BT of flexible nano-liposomes (mean ± SD, n = 3)

Formulations	Encapsulated efficiency (%)	Drug loading (%)
TRA-BT-FNL	98.74 ± 0.78	2.00 ± 0.04
TRA-FNL	99.02 ± 0.54	2.04 ± 0.15
TRA-BT-FNL	99.14 ± 0.20	1.95 ± 0.05
BT-FNL	98.89 ± 0.32	2.02 ± 0.03

### In vitro drug release

**Fig. 2:**
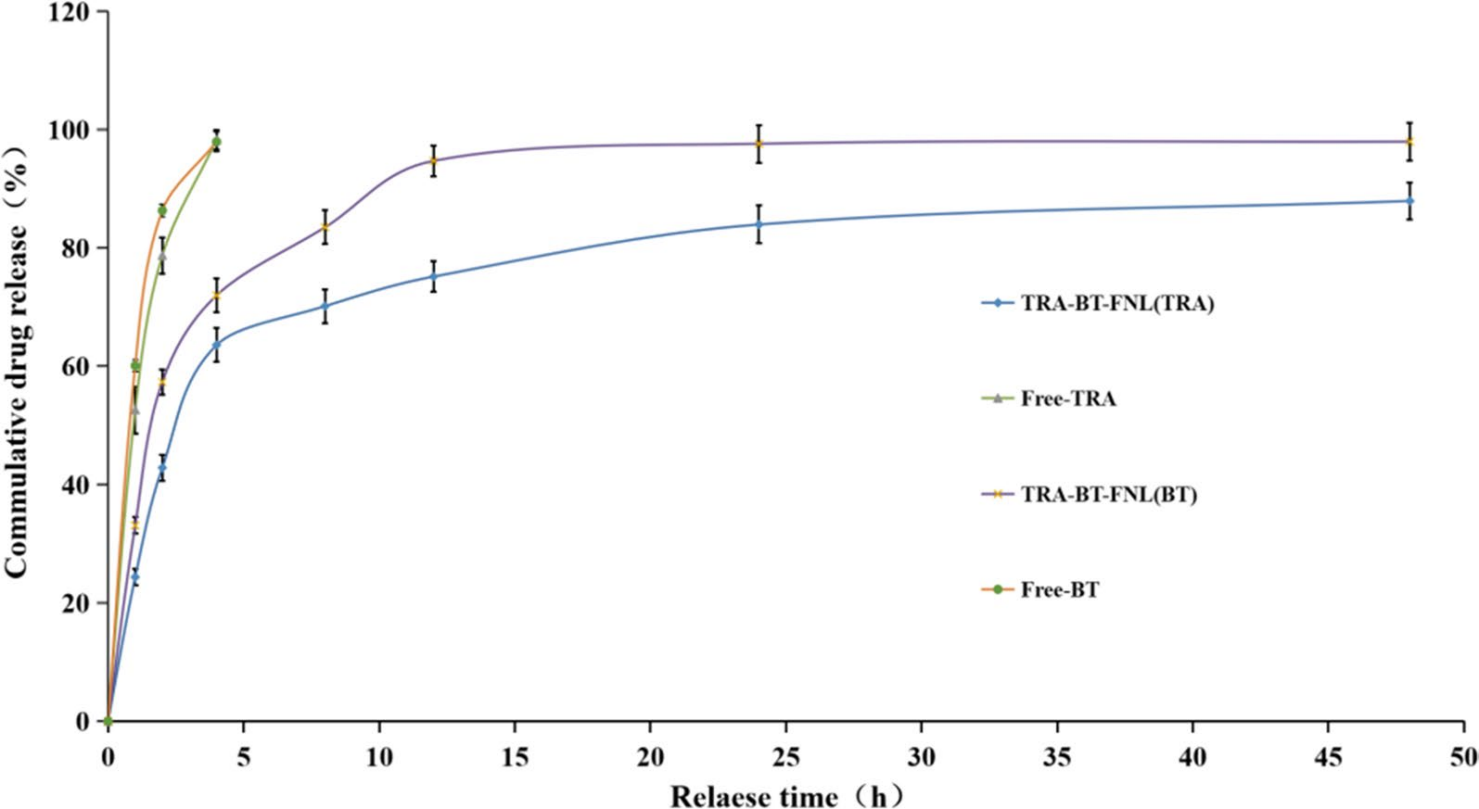
In vitro drug release behavior of free drugs and co-loaded flexible nano-liposomes (mean ± SD, n = 3)

In vitro drug release behaviors of different formulations were carried out under aqueous ethanol solution (pH 6.8, 37 °C). As shown in Fig. 2, both free TRA and BT were rapidly released, with nearly 100% of TRA or BT was released within 4 h. In contrast, both TRA and BT displayed sustained drug release behaviors when they were encapsulated within liposomes, although a relatively fast release rate was observed in the initial 12 h. The sustained drug release was attributed to that the coating layers of liposomes act as a barrier to protect the drugs from release. This behavior has advantages of reducing the widespread distribution of the drug after its use, allowing more drugs to be accumulated in the target sites for a better therapy.

### In vitro skin permeation and retention

**Fig. 3:**
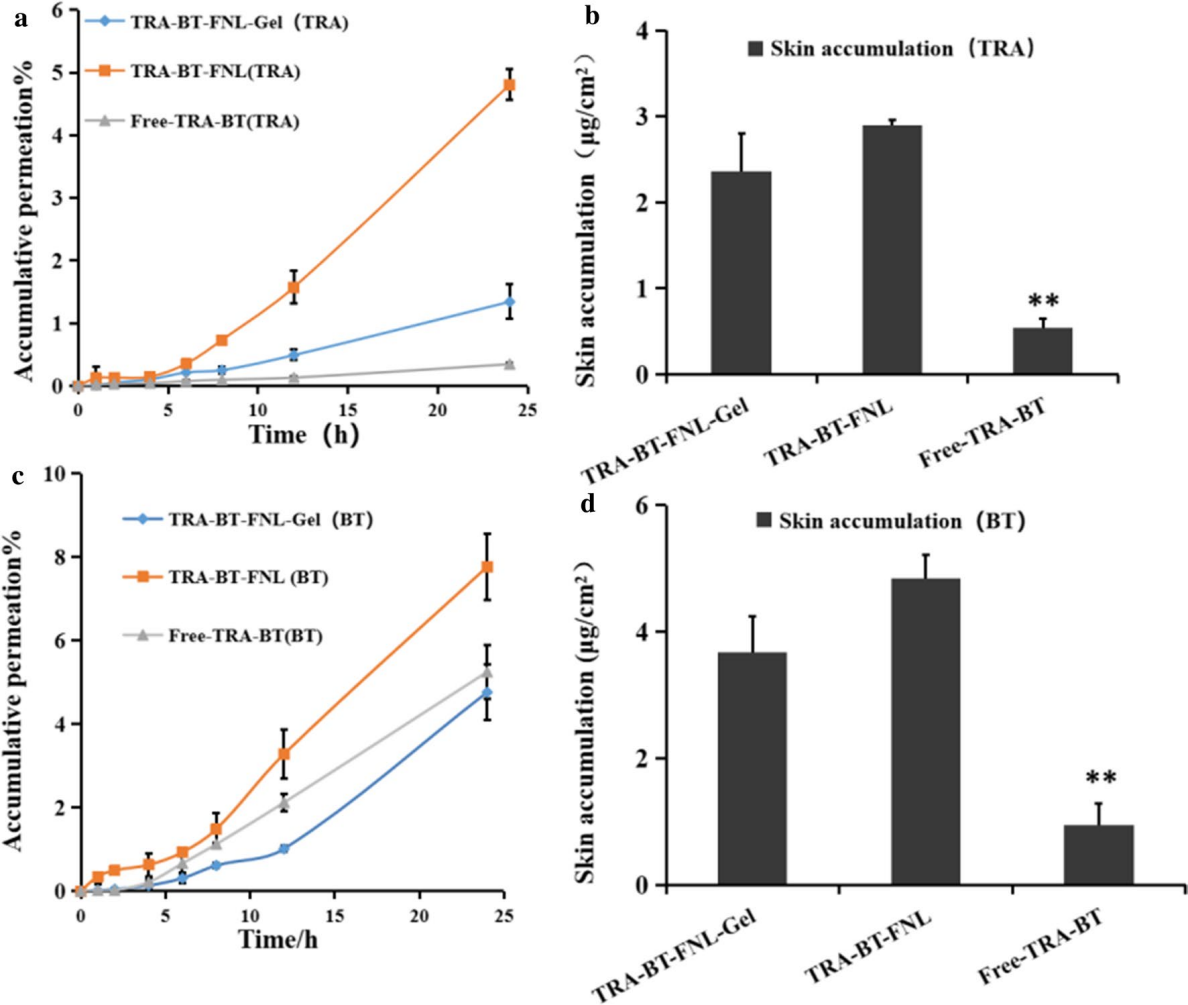
In vitro skin permeation and retention study of free drug, flexible nano-liposomes and liposomal gel: **a** The permeation profile of TRA in different formulations; **b** skin retention of TRA at 24 h; **c** the permeation profile of BT in different formulations; **d** the skin retention of BT at 24 h. (mean ± SD, n = 3; ^*^p < 0.05, ^**^p < 0.01, compared with TRA-BT-FNL group)

In vitro skin permeation and retention of different formulations were performed based on Franz diffusion cells, with the corresponding results illustrating in Fig. 3. As shown in Fig. 3a and c, total amounts of drugs (TRA or BT) accumulated in the receptor chambers were increased with prolonged incubation times, suggesting that the skin permeation of TRA and BT had time-dependent manners, regardless of the formulation type. However, the transdermal efficiency of the drugs highly depended on the formulation type. It was found that TRA in TRA-BT-FNL could more easily pass through the skin layer than that in free TRA and TRA-BT-FNL-Gel, suggesting that flexible liposomes possess the best transdermal efficiency for encapsulated TRA. Results for the skin retention of different formulation suggested that skin retention of TRA in the TRA-BT-FNL group was 1.23 and 5.40 times higher than that of TRA-BT-FNL-Gel and Free-TRA-BT group, respectively (Fig. 3b). Similar tendency was also observed for the skin permeation and retention of BT-loaded formulations (Fig. 3d). It is well known that the stratum corneum of skin acts a barrier to inhibit the skin permeation and retention of different drugs, such as TRA and BT [12]. The higher skin permeation and retention of drugs observed in flexible liposomes could be explained by a number of reasons [8, 42, 43]: (i) flexible liposomes with nano-scaled particles can closely contact with the surface of skin, thus facilitating the drug to permeate across the skin; (ii) the phospholipid molecules can integrate with the lipid covering of the stratum corneum, acting as drug penetration enhancer to conquer the barrier function of the skin; (iii) liposomes offer drug occlusive property, which contributes to a reduction in trans-epidermal water loss. In addition, the relative higher viscosity of liposomal gel matrix may be responsible for the reduction of drug skin permeation and retention occurring in liposomal gel. This result was agreed with the trend previously report in ointment [12], which is beneficial in reducing the frequency of application.

### In vitro cytotoxicity and cellular uptake

**Fig. 4:**
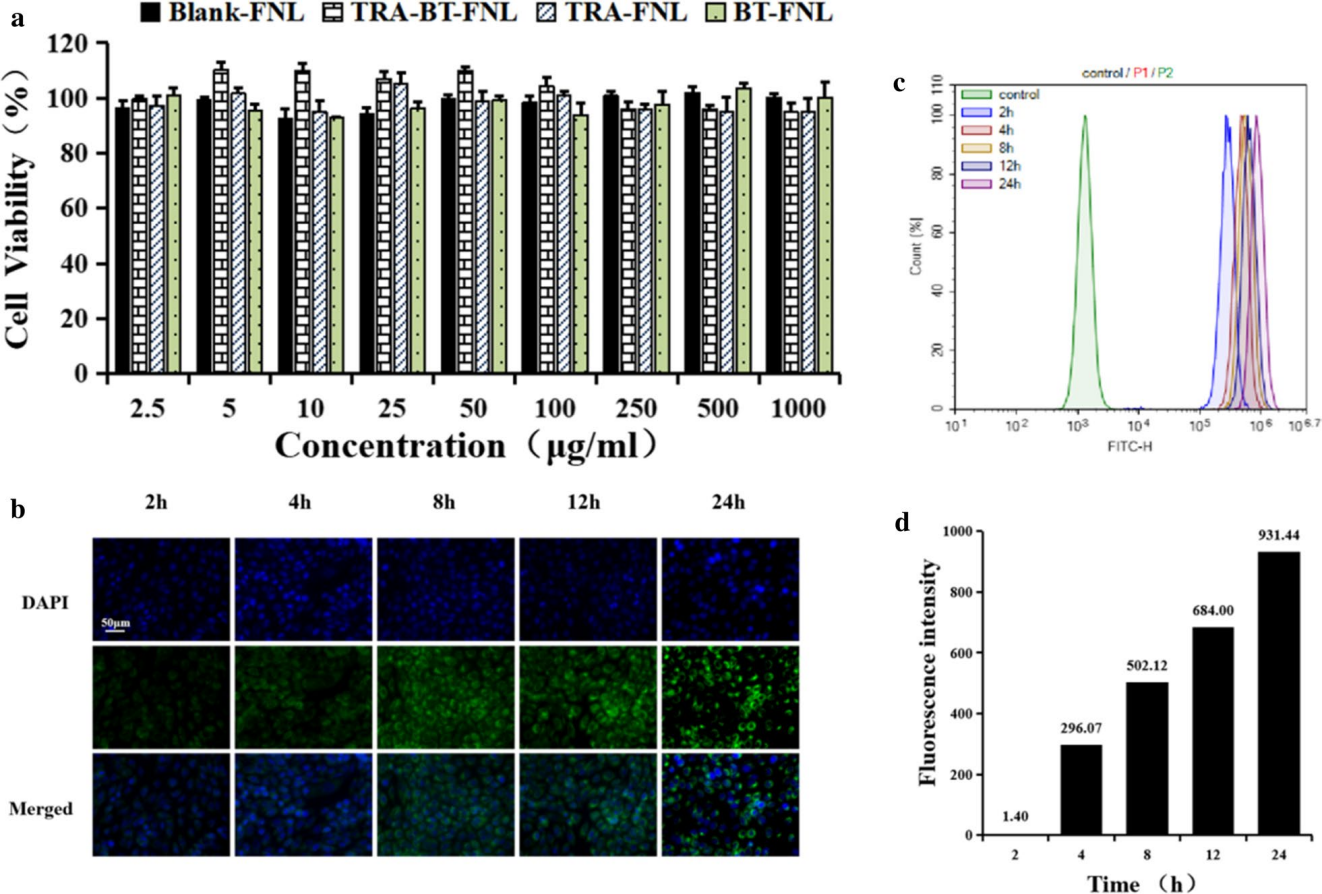
In vitro cytotoxicity and cellular uptake of flexible liposome: **a** Cytotoxicity of flexible liposomes towards HaCaT cell as a function of concentration; **b** fluorescence microscopy images of HaCaT cells after incubation with ODA-FITC-labeled flexible liposomes. **c** Fluorescent intensity signal of HaCaT cells after incubation with ODA-FITC-labeled flexible liposomes. **d** Fluorescence intensity value after incubation with ODA-FITC-labeled flexible liposomes

In vitro cytotoxicity of flexible liposomes encapsulated with or without drug towards HaCaT cells were investigated via the MTT assay. It was observed that all formulations were little toxic to the cells at tested concentrations of 2.5–1000 μg/mL, as evidence showing that the cell viability were over 95% (Fig. 4a).

The cellular uptake of flexible liposomes was evaluated by a fluorescence microscope and flow cytometry using HaCaT cells (Fig. 4b–d). FITC, a commonly used fluorescent dye, was used to label the flexible liposomes before analysis. Fluorescence microscope images exhibited that the green fluorescence signals of FITC increased with increasing the incubation times, indicating that the cellular uptake of flexible liposomes was time dependent. Flow cytometry examination also suggested that the fluorescence intensity of cells with 24 h of incubation was higher than the other groups, further confirming the time-dependent cellular uptake of flexible liposomes.

### In vivo skin distribution

**Fig. 5:**
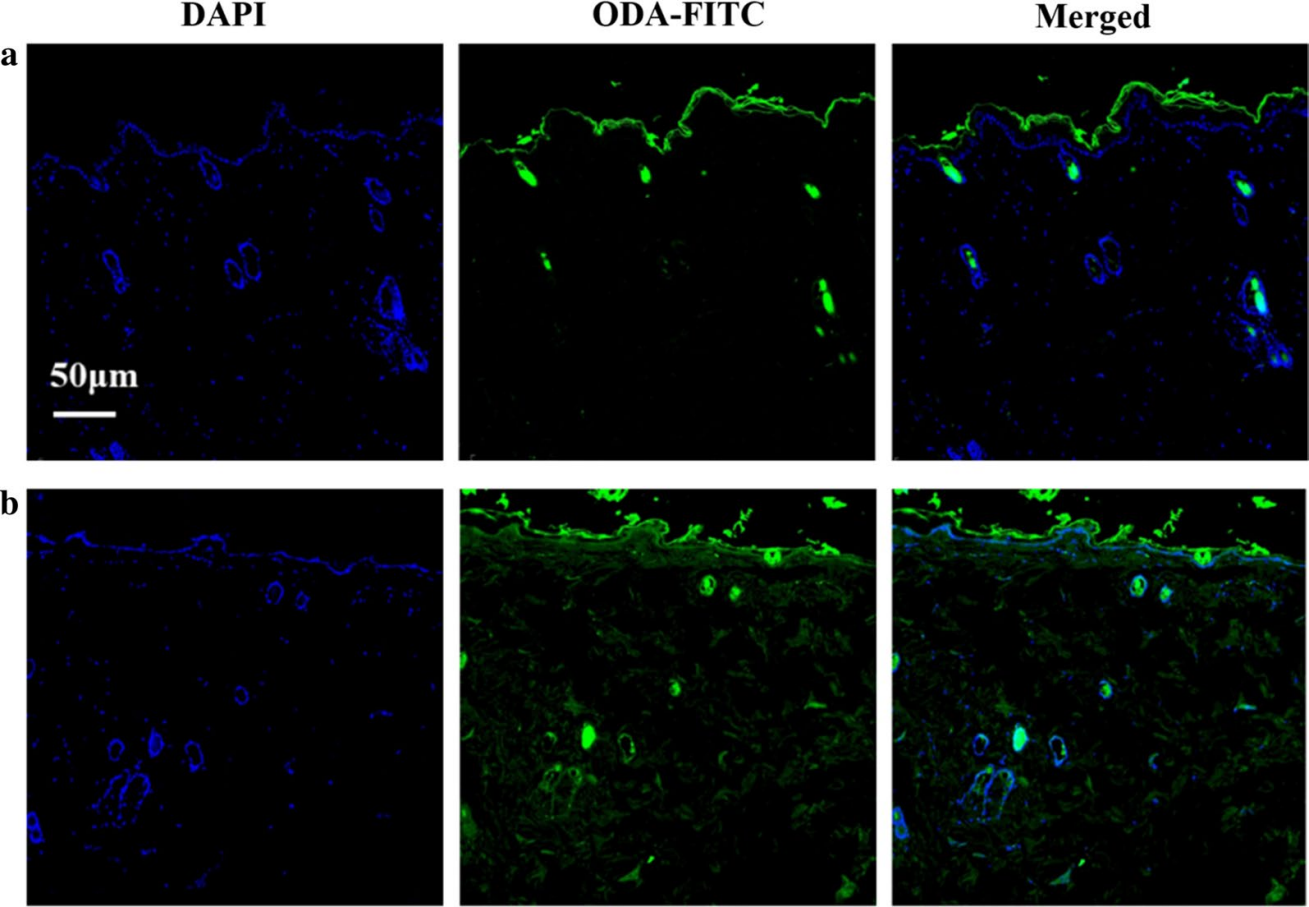
Fluorescence microscopy images of skin sections treated with ODA-FITC-labeled gel for 12 h: **a** ODA-FITC-gel; **b** ODA-FITC-FNL gel

In vivo skin distribution of gel containing free drug or drug-loaded liposomes was investigated by a confocal laser scanning microscope. Here, ODA-FITC was used as a fluorescent probe for visible observation of dermal distribution. As shown in Fig. 5, ODA-FITC-loaded liposomal gel resulted in a greater penetration than the other one. This behavior was related to the penetration enhancement effect of liposomal formulation, which was also discussed in section “In vitro skin permeation and retention”.

### Establishment of psoriatic animal models

**Fig. 6:**
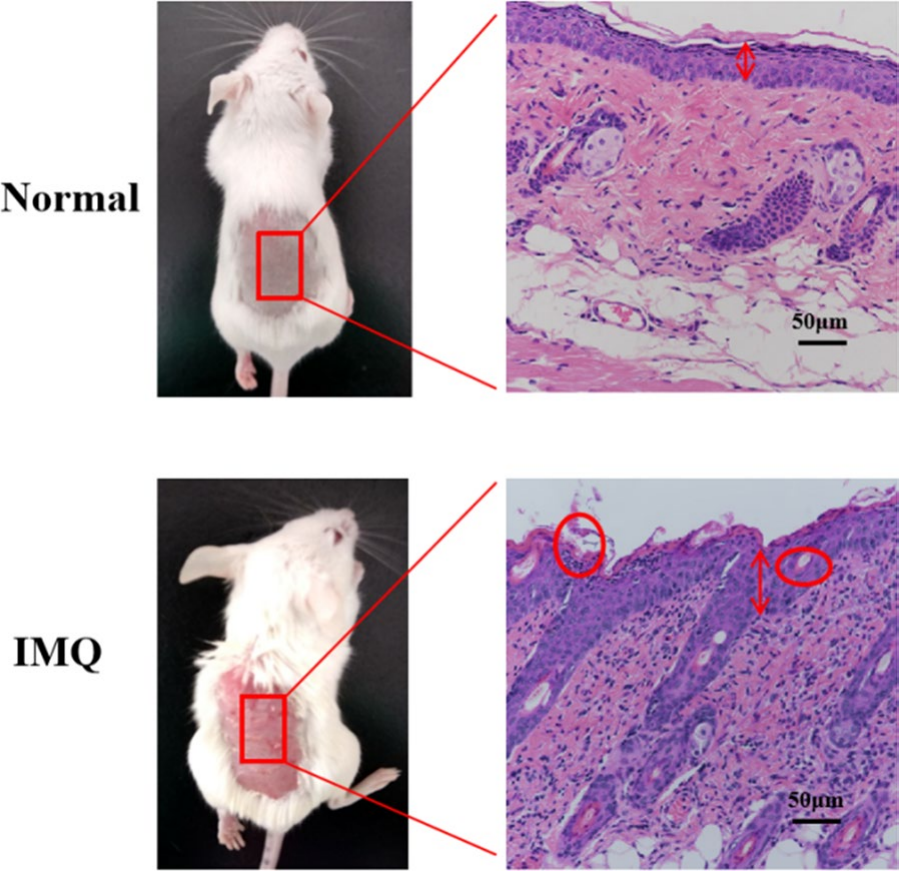
Representative photos and H&E staining of skin treated with or without IQM for 7 days

The establishment of psoriatic animal models was performed by inducing the skin of mice with IQM. After 7 days of inducing with IQM, the back skin of mice exhibited plaque with marked erythema and scaling, which is the phenotypic characters of psoriasis (Fig. 6). In addition, the histopathological changes in the skin of IQM-induced mice were examined by using H&E-stained skin sections. It was found that IQM-induced skin revealed the characteristics of increased epidermal proliferation, abnormal differentiation, epidermal accumulation of neutrophils in microabscesses, which is the histopathological features of psoriatic skin [44]. These results demonstrated that psoriatic animal models were successfully built in our study, which were then used in the following experiments.

### Anti-psoriatic efficiency

#### Scoring severity of skin inflammation

The effect of liposomal gel type on anti-psoriatic efficiency was firstly evaluated by scoring the severity of erythema and skin thickening. In our study, PASI scores of erythema and skin thickening in the psoriatic skin area were recorded on day 3, day 5 and day 7 (Fig. 8a–c). It was found that all formulations displayed positive scores for both of erythema and skin thickening at day 3, which was progressively increased at a prolonged time of day 7. As compared with IMQ, psoriatic skin treated with TRA-BT-FNL-Gel, TRA-FNL-Gel, BT-FNL-Gel, Free-TRA-BT-Gel, Free-TRA-Gel or Free-BT-Gel showed reductions in both skin thickening and erythema to different extent at day 7. Looking closely, the scores of TRA-BT-FNL-Gel groups were significantly lower than those of other groups, indicating that TRA-BT-FNL-Gel has the best anti-psoriatic ability.

**Fig. 7:**
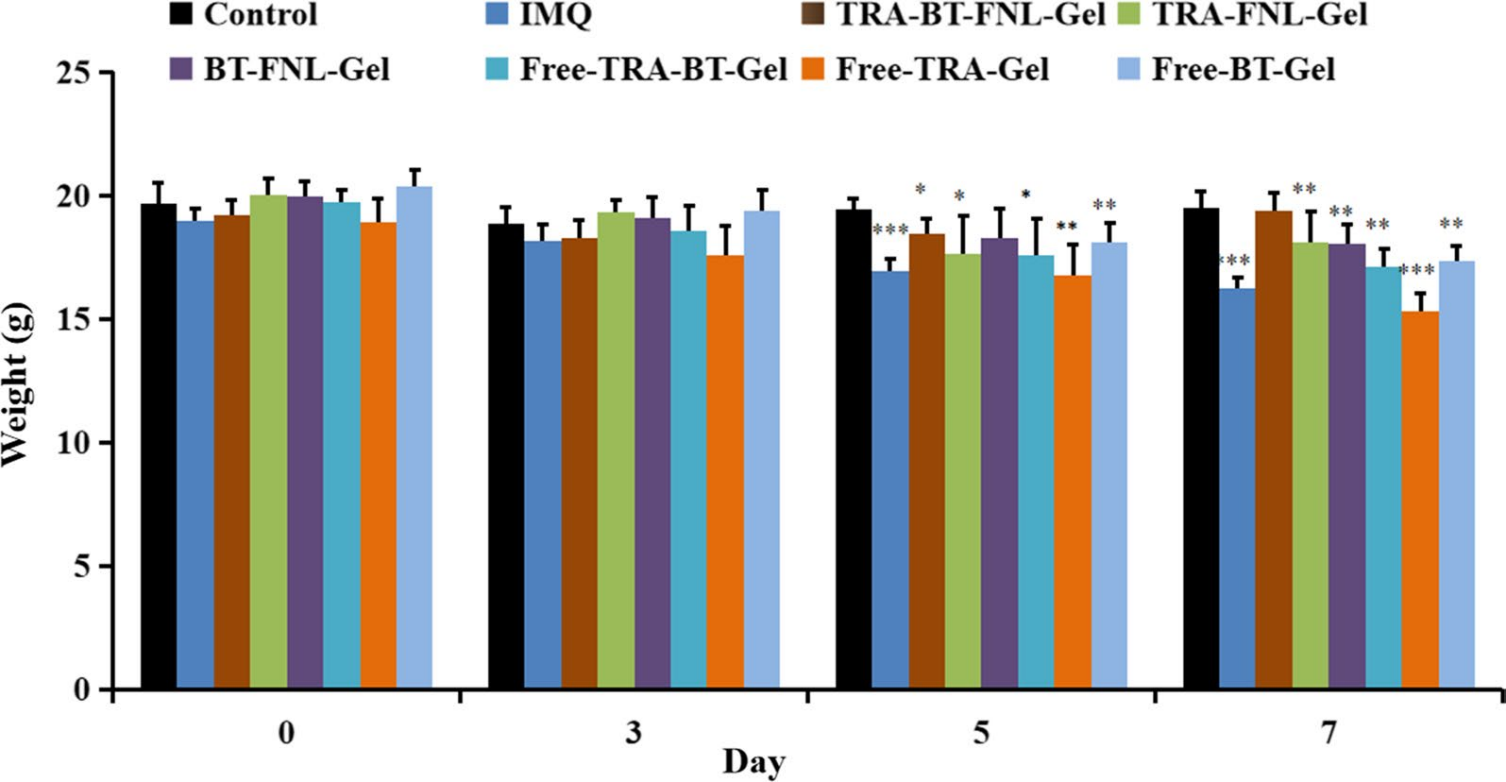
Weight changes of mice in different groups (mean ± SD, n = 5; ^*^p < 0.05, **p < 0.01, ***p < 0.001 compared with the weight of control group)

**Fig. 8:**
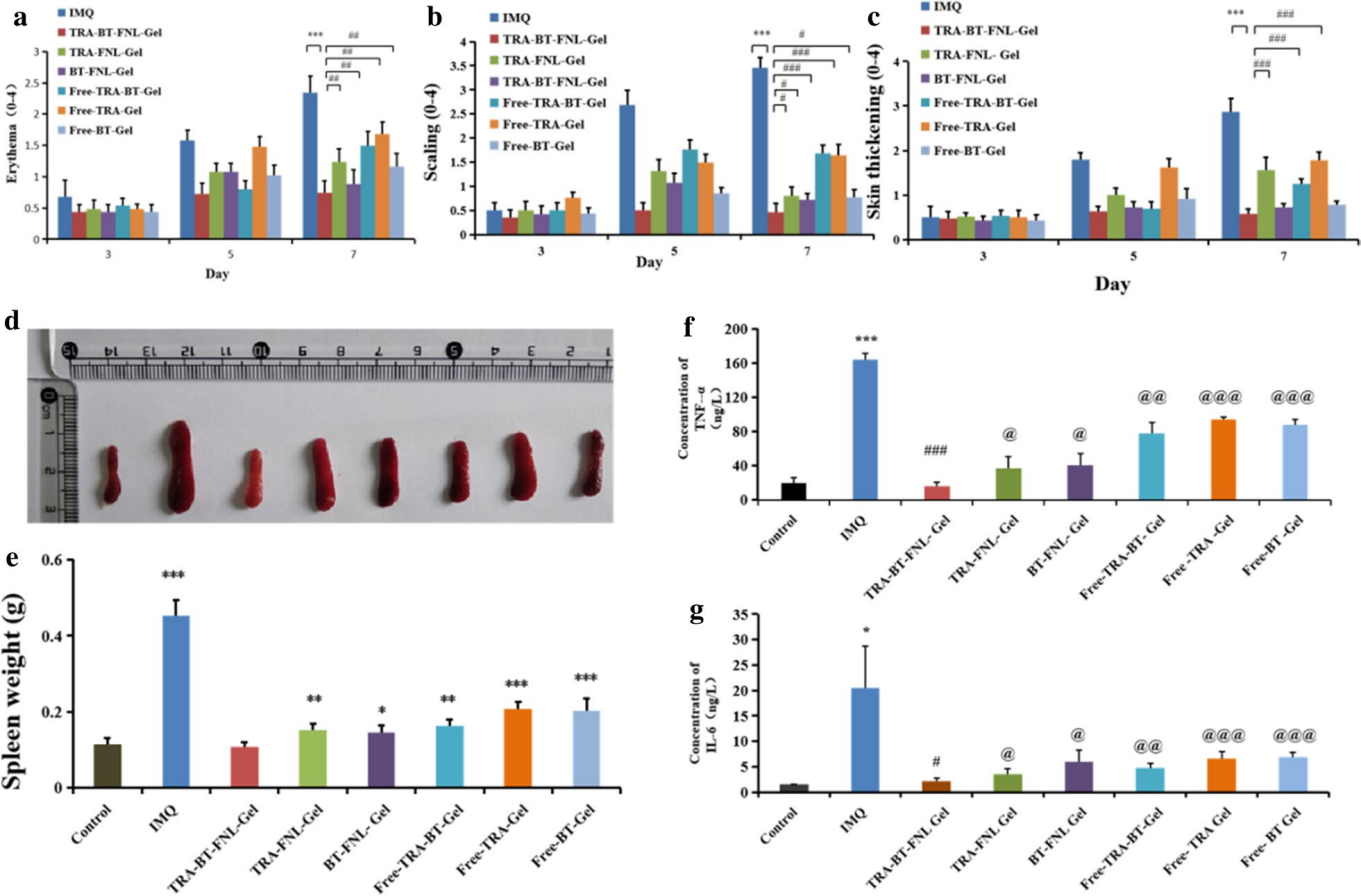
Anti-psoriasis pharmacodynamics. **a** PASI scoring for erythema; **b** PASI scoring for scaling; **c** PASI scoring for skin thickening (mean ± SD, n = 5; ^*^p < 0.05, ^**^p < 0.01, ^***^p < 0.001 compared with IMQ group; ^#^p < 0.05, ^##^p < 0.01, ^###^p < 0.001 compared with TRA-BT-FNL-Gel group; **d** representative photos of spleens in different group; **e** the changes of spleen weight in different groups, (mean ± SD, n = 5; ^*^p < 0.05, **p < 0.01, ***p < 0.001 compared with the weight of control group); **f** expression levels of TNF-α in different group,(g) Expression levels of IL-6 in in different group, (mean ± SD, n = 5; ^*^p < 0.05, ^**^p < 0.01, ^***^p < 0.001 compared with control group; ^#^p < 0.05, ^##^p < 0.01, ^###^p < 0.001 compared with IMQ group, ^@^p < 0.05, ^@@^p < 0.01, ^@@@^p < 0.001 compared with TRA-BT-FNL-Gel group

The change in body weights of mice treated with different formulations were recorded during 7 days of the experiment. As shown in Fig. 7, the body weights of IQM and Free-TRA-Gel groups were significantly reduced after 7 days of treatment. The reduction of body weights was possibly due to the adverse reactions of IQM and TRA. On the other hand, the body weights were little changed in TRA-FNL-Gel group, which could be attributed to the reduced side effects endowed by liposomal encapsulation of TRA. The enlargement of spleen is an important indicator of immunological diseases, such as psoriasis [38]. The spleens collected from different groups were therefore photographed and weighted (Fig. 8d and e). Apparently, the spleen of IQM-groups was much larger and heavier than other groups, suggesting that the treatment of drug-loaded liposomal gel has positive impacts on the psoriatic skin. It should be noted that the spleen weights of TRA-BT-FNL-Gel group was similar to the control group, showing the best ability to inhibit spleen enlargement after IQM induction, which further confirmed its superior effect of anti-psoriasis.

#### Histopathology examination and immunohistochemical analysis

**Fig. 9:**
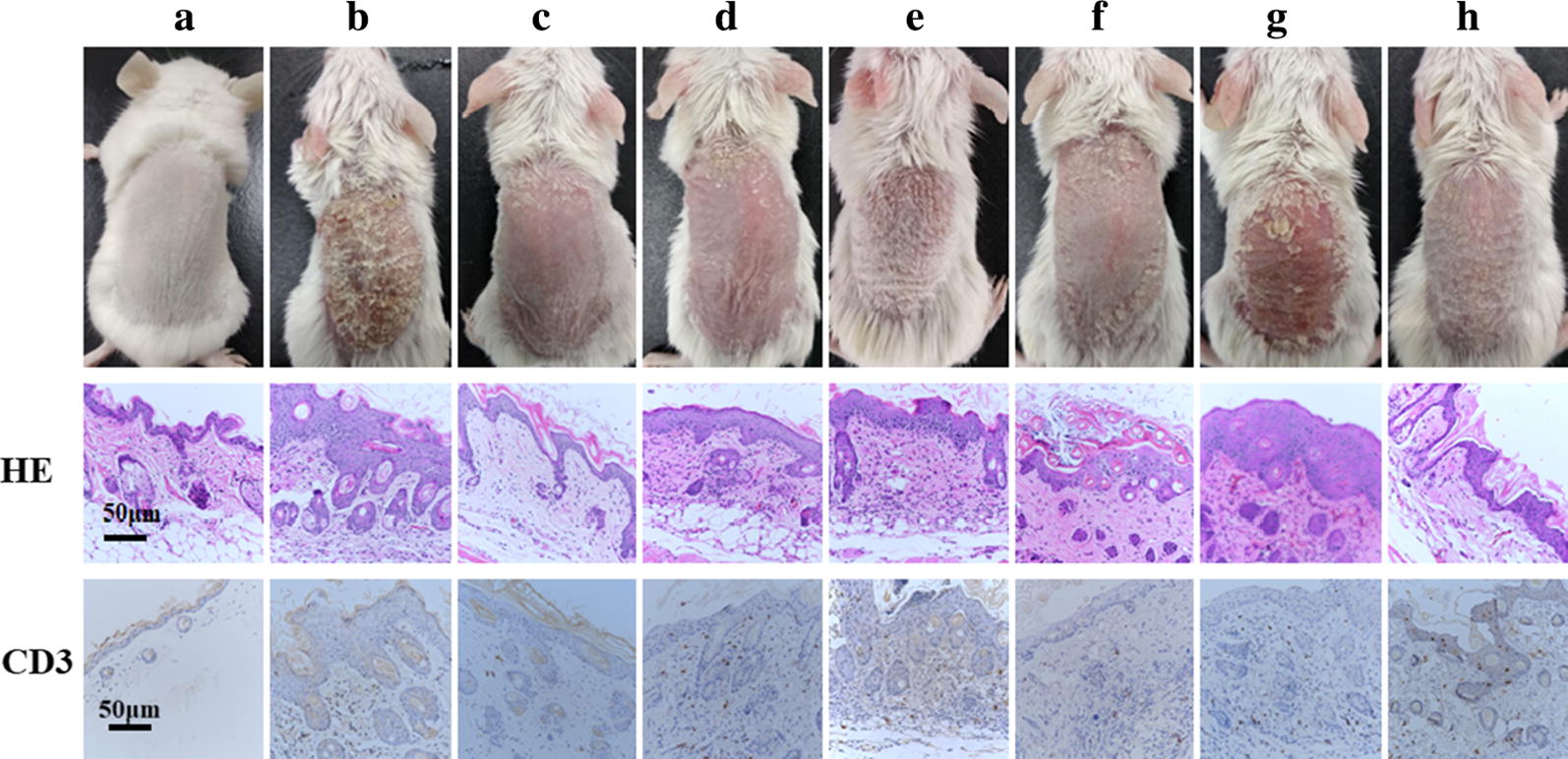
H&E staining and CD3 expression of the dorsal skin phenotype and diseased tissue sections of mice after treating with different drugs (**a** Control; **b** IMQ; **c** TRA-BT-FNL-Gel; **d** TRA-FNL-Gel; **e** BT-FNL-Gel; **f** Free-TRA-BT-Gel; **g** Free-TRA-Gel; **h** Free-BT-Gel)

The effect of different treatment on the recovery of psoriatic skin was also investigated by H&E staining method (Fig. 9). Histopathological examination suggested the health rats showed normal skin characterized by regular epidermis and dermis. In comparison, the skin of IQM group had characteristic features of psoriasis, including the presence of hyperkeratosis, parakeratosis, and epidermal infiltrates. The presence of acanthosis and increased infiltration of leukocytes were also observed in the IMQ-induced skin. The histopathological changes of treatment groups were highly dependent on the formulation type. After treated with Free-TRA-Gel, psoriatic skin showed slight reduction the thickness of epidermis, with obvious evidence of acanthosis being observed. This result suggested that the anti-psoriatic efficiency of Free-TRA-Gel was quite limited, which was possibly due to the skin irradiation of TRA. In contrast, TRA-FNL-Gel exhibited a better ability to reduce the thickness of epidermis, indicating that encapsulation of TRA within liposomes could increase the anti-psoriatic effect, which was due to reduced side effects. After treating with TRA-FNL-Gel, BT-FNL-Gel or Free-TRA-BT-Gel, acanthosis was observed but the extent of severity was less than IMQ group. It should be noted only TRA-BT-FNL-Gel group showed similar histopathological characteristics in comparison to the control group, where the epidermis of the skin was almost normalized and less infiltrates were observed. In addition, no sign of acanthosis was observed.

The expression level of CD3 in psoriatic skin is also an important parameter to understand the anti-psoriatic efficacy of different formulation. Results on immunohistochemistry suggested that there was a large amount of CD3 expression on the skin of IMQ group, which was in line with those reported in previous studies [45, 46]. In contrast, the expression level of CD3 was significantly decreased in the treatment group, in which TRA-BT-FNL gel group showed the lowest level. This result indicated that TRA-BT-FNL gel had the best anti-psoriatic efficacy than other ones.

#### Cytokines determination

The cytokines of TNF-α and IL-6 play an important role in the development of psoriasis [47]. The levels of TNF-α and IL-6 in psoriatic skin were determined by ELISA assay. As shown in Fig. 8f and g, in comparison with the control group, The IOM group showed that the levels of TNF-α and IL-6 were increased by 8.56 and 14.01 times, respectively. It was also observed that the levels of TNF-α was decreased by 4.43, 4.05, 2.12, 1.75, and 1.78 times after the psoriatic skin was treated with TRA-BT-FNL-Gel, TRA-FNL-Gel, BT-FNL-Gel, Free-TRA-BT-Gel, Free-TRA-Gel, and Free-BT-Gel, respectively (Fig. 8f). Similar tendency was also observed in the results for the levels of IL-6 (Fig. 8g). The highest anti-psoriatic activity of TRA-BT-FNL-Gel might be responsible for its best capability of reducing the cytokine levels of TNF-α and IL-6.

### Skin irritation

**Fig. 10:**
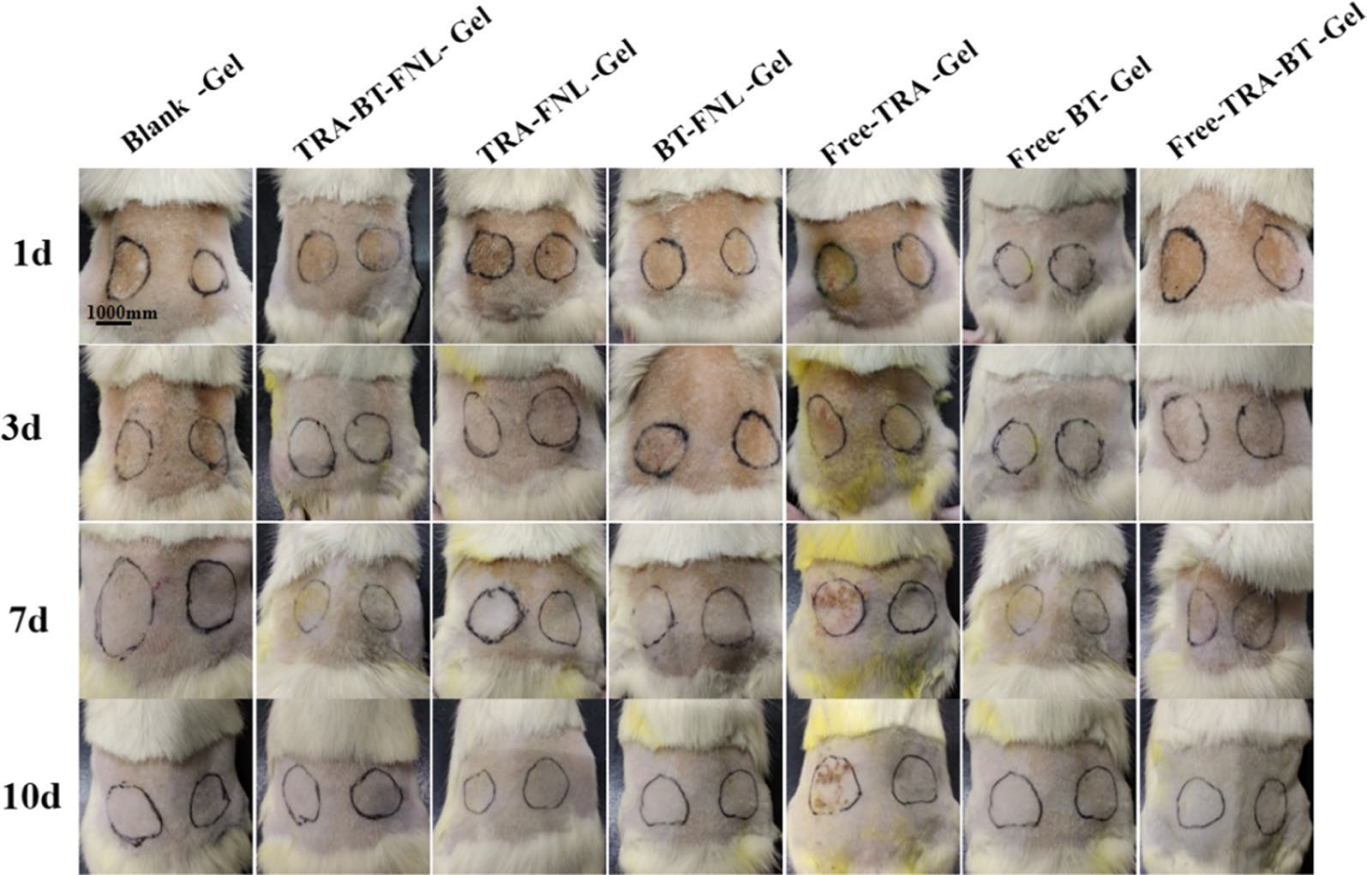
Representative photos of skin treated with different formulations at various time point (left: drug loading gel group, right: blank gel group)

Skin compliance study of liposomal gels was performed on the skin of healthy Wistar rats (Fig. 10 and Table 3). It was found that the utilization of free TRA could cause skin irradiation. In contrast, evidence of skin irradiation was not observed in the other groups. This result confirmed that the skin toxicity caused by long-term administration of TRA could considerably decreased by encapsulating this drug in liposomal gel. Previous study also suggested that liposomal gel is effective at reducing the side effects of the drugs, such as tacrolimus [38]. This result suggested that TRA-BT-FNL gel is safe for topical application.

**Table 3:** Score of each prescription gel normal skin stimulation experiment (mean ± SD, n = 6)

Formulations	24 h	3 days	7 days	10 days
Blank-Gel	0	0	0	0
TRA-BT-FNL- Gel	0	0	0	0
TRA -FNL- Gel	0	0	0	0
BT-FNL- Gel	0	0	0	0
Free- TRA -Gel	1.33 ± 0.26	1.62 ± 0.14	2.67 ± 0.13	2.38 ± 0.14
Free- BT- Gel	0	0	0	0
Free-TRA-BT -Gel	0	0	0	0

## Conclusions

In this work, a liposomal gel containing combination of TRA and BT was developed as a novel topical formulation for the treatment of psoriasis. TRA and BT dual-loaded flexible liposomes had average particle diameters of ~ 70 nm and high drug encapsulation efficiency of > 98%. Flexible liposomes offered the encapsulated drugs with an enhanced the skin permeation and retention. Cellular studies displayed that the dual-loaded flexible liposomes were nontoxic, and the cellular uptake was time-dependent. Flexible liposomes were then incorporated within the gel for convenience of application. In vivo studies revealed that TRA-BT liposomal gel could largely improve the histopathological features of IMQ-induced skin, as well as reduce the levels of TNF-α and IL-6. Studies also indicated that TRA-BT liposomal gel had an enhanced anti-psoriatic efficacy than BT or TRA liposomal gel alone. The results obtained in our study suggested TRA-BT liposomal gel has the potential to be a desirable formulation for treatment of psoriasis.

## Supplementary information

**Additional file 1: Figure S1.** The change of TRA degradation rate over the time (a) Photostability; (b) Oxygen stability.

## Data Availability

All data generated or analyzed during this study are included in this article and its additional file.

## Abbreviations

TRA: All trans-retinoic acid
BT: Betamethasone
FNL: Flexible liposomes
TEM: Transmission electron microscopy
TNF-α: Tumor necrosis factor-α
IL-6: Interleukin-6
IL-8: Interleukin-8
PASI: Psoriasis Area And Severity Index
IMQ: Imiquimod
FITC: Fluorescein isothiocyanate
ODA: Octadecylamine
EE: Encapsulation efficiency
DL: Drug loading
PDI: Polydispersity index
ELISA: Enzyme-linked immunosorbent assay
KC: Keratinocyte
GC: Glucocorticoid

## References

[1] Gudjonsson J (2004). Immunopathogenic mechanisms in psoriasis. Clin Exp Immunol.

[2] Takeshita J (2017). Psoriasis and comorbid diseases: epidemiology. J Am Acad Dermatol.

[3] Say M (2018). Clinical and therapeutic aspects of linear psoriasis: a study of 30 cases. Am J Clin Dermatol.

[4] Cornell RC (1992). Clinical trials of topical corticosteroids in psoriasis: correlations with the vasoconstrictor assay. Int J Dermatol.

[5] Gelfand JM (2012). Comparative effectiveness of commonly used systemic treatments or phototherapy for moderate to severe plaque psoriasis in the clinical practice setting. Arch Dermatol.

[6] Dai H (2018). Restoration of CD3+ CD56+ cell level improves skin lesions in severe psoriasis: a pilot clinical study of adoptive immunotherapy for patients with psoriasis using autologous cytokine-induced killer cells. Cytotherapy.

[7] Lassus A (1980). Systemic treatment of psoriasis with an oral retinoic acid derivative (Ro 10‐9359). Br J Dermatol.

[8] Pradhan M (2018). Understanding the prospective of nano-formulations towards the treatment of psoriasis. Biomed Pharmacother.

[9] Pradhan M, Singh D, Singh MR (2016). Influence of selected variables on fabrication of Triamcinolone acetonide loaded solid lipid nanoparticles for topical treatment of dermal disorders. Artif Cells Nanomed Biotechnol.

[10] Layton AM, Cunliffe WJ (1992). Guidelines for optimal use of isotretinoin in acne. J Am Acad Dermatol.

[11] Raza K (2013). Nano-lipoidal carriers of tretinoin with enhanced percutaneous absorption, photostability, biocompatibility and anti-psoriatic activity. Int J Pharm.

[12] Lu K-J (2019). A dual deformable liposomal ointment functionalized with retinoic acid and epidermal growth factor for enhanced burn wound healing therapy. Biomater Sci..

[13] Demetriou A (1985). Vitamin A and retinoic acid: induced fibroblast differentiation in vitro. Surgery.

[14] Zouboulis CC (2001). Retinoids–which dermatological indications will benefit in the near future?. Skin Pharmacol Physiol.

[15] Kitamura H (2007). Cytokine modulation of retinoic acid-inducible gene-I (RIG-I) expression in human epidermal keratinocytes. J Dermatol Sci.

[16] Gottlieb AB (1986). Expression of HLA-DR molecules by keratinocytes, and presence of Langerhans cells in the dermal infiltrate of active psoriatic plaques. J Exp Med.

[17] Chen J et al. Formulation and evaluation of a topical liposomal gel containing a combination of zedoary turmeric oil and tretinoin for psoriasis activity. J Liposome Res, 2020(just-accepted): p. 1–47. 10.1080/08982104.2020.1748646 32223352

[18] Rahman SA (2015). Formulation of tretinoin-loaded topical proniosomes for treatment of acne: in vitro characterization, skin irritation test and comparative clinical study. Drug Delivery.

[19] Saki N (2018). Comparing the efficacy of triamcinolone acetonide iontophoresis versus topical calcipotriol/betamethasone dipropionate in treating nail psoriasis: a bilateral controlled Clinical Trial. Dermatol Res Pract.

[20] Uva L (2012). Mechanisms of action of topical corticosteroids in psoriasis. Int J Endocrinol.

[21] Yamauchi P (2017). DFD-01: a novel topical formulation of betamethasone dipropionate for the treatment of extensive psoriasis. Expert Rev Clin Immunol.

[22] Sarkar MK (2017). Endogenous glucocorticoid deficiency in psoriasis promotes inflammation and abnormal differentiation. J Investig Dermatol.

[23] Mori H (2016). Effects of topical application of betamethasone on imiquimod-induced psoriasis-like skin inflammation in Mice. Kobe J Med Sci.

[24] Martello LA (2000). Taxol and discodermolide represent a synergistic drug combination in human carcinoma cell lines. Clin Cancer Res.

[25] Coman G (2017). A randomized, split-face, controlled, double-blind, single-centre clinical study: transient addition of a topical corticosteroid to a topical retinoid in patients with acne to reduce initial irritation. Br J Dermatol.

[26] Xia L (2018). Efficacy, safety, and cost-effectiveness of all-trans retinoic acid/Clobetasol propionate compound ointment in the treatment of mild to moderate psoriasis vulgaris: a randomized, single-blind, multicenter clinical trial. Dermatol Ther.

[27] Kaidbey KH, Petrozzi JW, Kligman AM (1975). Treatment of psoriasis with topically applied tretinoin and steroid ointment. Arch Dermatol.

[28] Macdonald A, Fry L (1972). Retinoic acid in the treatment of psoriasis. Br J Dermatol.

[29] Hua S (2015). Lipid-based nano-delivery systems for skin delivery of drugs and bioactives. Front Pharmacol.

[30] Li W-Z (2016). Propylene glycol-embodying deformable liposomes as a novel drug delivery carrier for vaginal fibrauretine delivery applications. J Control Release.

[31] Badran M, Shalaby K, Al-Omrani A (2012). Influence of the flexible liposomes on the skin deposition of a hydrophilic model drug, carboxyfluorescein: dependency on their composition. Sci World J.

[32] Yang C (2019). Coarse-grained molecular dynamics simulations of the effect of edge activators on the skin permeation behavior of transfersomes. Colloids Surf B.

[33] Jiang T (2018). Enhanced transdermal drug delivery by transfersome-embedded oligopeptide hydrogel for topical chemotherapy of melanoma. ACS Nano.

[34] Wasankar SR, Faizi SM, Deshmuk AD (2012). Formulation and development of liposomal gel for topical drug delivery system. Int Jf Pharm Sci Res.

[35] Zhang Y (2018). Formulation and in vitro stability evaluation of ethosomal carbomer hydrogel for transdermal vaccine delivery. Colloids Surf B.

[36] dos Santos, RS et al. The effect of carbomer 934P and different vegetable oils on physical stability, mechanical and rheological properties of emulsion-based systems containing propolis. J Mol Liq, 2020: p. 112969.

[37] Li S-J (2017). Targeting delivery of simvastatin using ICAM-1 antibody-conjugated nanostructured lipid carriers for acute lung injury therapy. Drug Delivery.

[38] Jain A (2016). Tacrolimus and curcumin co-loaded liposphere gel: synergistic combination towards management of psoriasis. J Control Release.

[39] van der Fits L (2009). Imiquimod-induced psoriasis-like skin inflammation in mice is mediated via the IL-23/IL-17 axis. J Immunol.

[40] Verma DD (2003). Particle size of liposomes influences dermal delivery of substances into skin. Int J Pharm.

[41] Shimizu K (2003). Stability and antitumor effects of all-trans retinoic acid-loaded liposomes contained sterylglucoside mixture. Int J Pharm.

[42] Alkilani AZ, McCrudden MT, Donnelly RF (2015). Transdermal drug delivery: innovative pharmaceutical developments based on disruption of the barrier properties of the stratum corneum. Pharmaceutics.

[43] Dragicevic-Curic N (2009). Temoporfin-loaded liposomal gels: viscoelastic properties and in vitro skin penetration. Int J Pharm.

[44] Di Cesare A, Di Meglio P, Nestle FO (2009). The IL-23/Th17 axis in the immunopathogenesis of psoriasis. J Investig Dermatol.

[45] Nomura T (2019). Recombination-induced revertant mosaicism in ichthyosis with confetti and loricrin keratoderma. J Dermatol Sci.

[46] Ma J (2019). D-Pinitol ameliorates imiquimod-induced psoriasis like skin inflammation in a mouse model via the NF-κB Pathway. J Environ Pathol Toxicol Oncol.

[47] Gibellini L (2016). Anti-TNF-α drugs differently affect the TNFα-sTNFR system and monocyte subsets in patients with psoriasis. PLoS ONE.

