# Supplementary material for: Flexible liposomal gel dual-loaded with all-trans retinoic acid and betamethasone for enhanced therapeutic efficiency of psoriasis
Source: J Nanobiotechnology. 2020 May 24;18:80. doi: 10.1186/s12951-020-00635-0 (PMC7245867; doi:10.1186/s12951-020-00635-0)
Supplement: Supplementary file 1 — Additional file 1: Figure S1. The change of TRA degradation rate over the time (a) Photostability; (b) Oxygen stability. [file 12951_2020_635_MOESM1_ESM.docx]

**Additional Information**

**Flexible liposomal gel dual-loaded with all-trans retinoic acid and betamethasone for enhanced therapeutic efficiency of psoriasis**

Wei Wang^1#^, Gao-feng Shu ^2#^, Kong-jun Lu ^2^, Xiao-ling Xu^2^, Min-cheng Sun^2^, Jing Qi^2^, Qiao-ling Huang^1*^, Wei-qiang Tan^3*^, Yong-zhong Du^2*^ ;

^1^The Third People's Hospital of Hangzhou, Hangzhou, 310009, China;

^2^ Institute of Pharmaceutics, College of Pharmaceutical Sciences, Zhejiang University, Hangzhou ,310058, China;

^3^ Department of Plastic Surgery, Sir Run Run Shaw Hospital, Zhejiang University School of Medicine, Hangzhou, 310058, China.

^#^: These authors contributed equally to this work.

*: Correspondence should be addressed to the following:

Qiao-ling Huang, Department of Pharmaceutics, The Third People's Hospital of Hangzhou, Hangzhou ,310009, China. E-mail: hql6512@163.com.

Wei-qiang Tan, M.D, Department of Plastic Surgery, Sir Run Run Shaw Hospital, Zhejiang University School of Medicine, Hangzhou,310016, China. E-mail: tanweixxxx@zju.edu.cn.

Dr. Yong-zhong Du, Institute of Pharmaceutics, College of Pharmaceutical Sciences, Zhejiang University, Hangzhou, 310058, China. E-mail: [duyongzhong@zju.edu.cn](mailto:duyongzhong@zju.edu.cn).

**
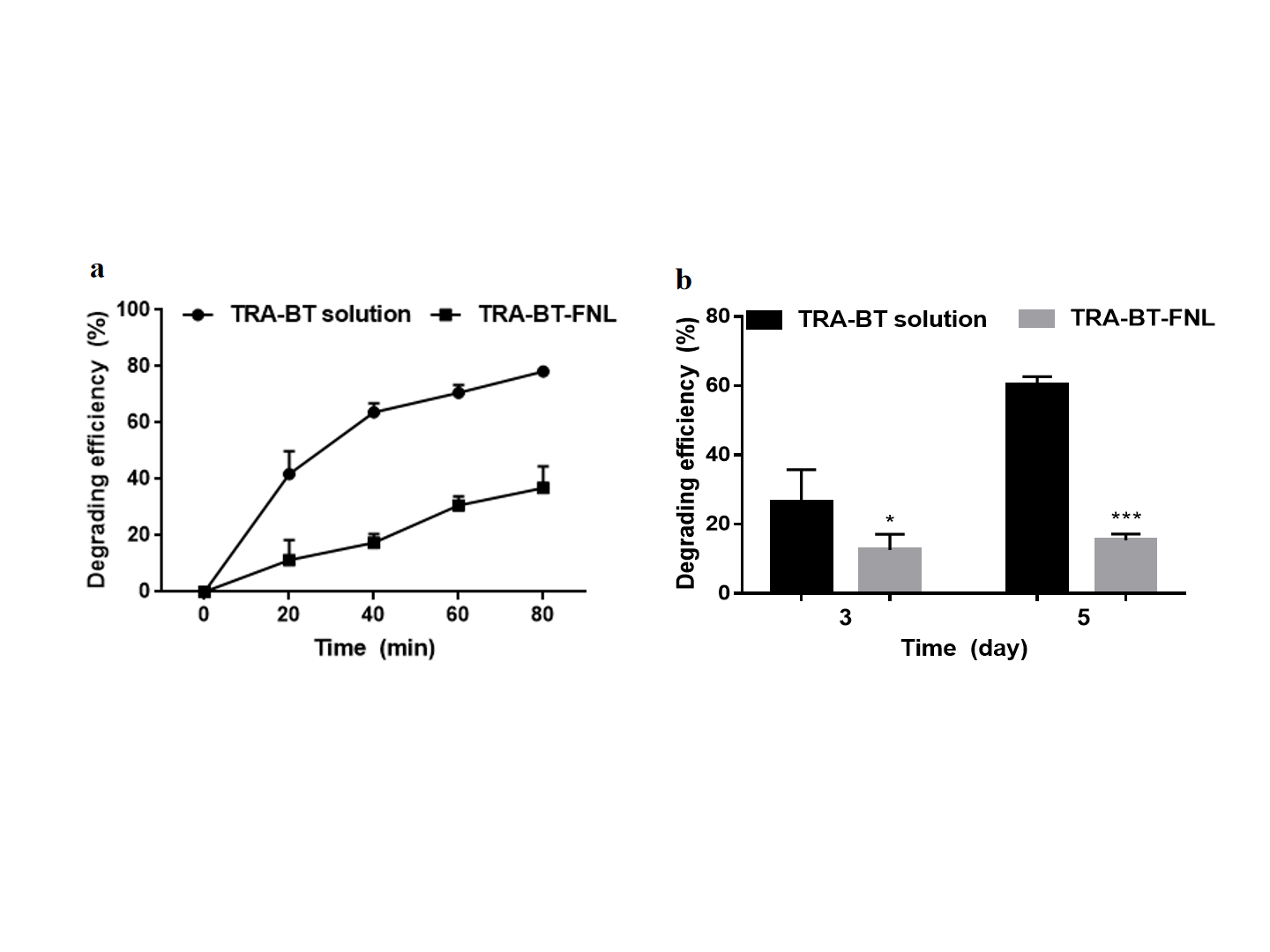
**

**Figure S1:** The change of TRA degradation rate over the time (a) Photostability; (b) Oxygen stability(mean ±SD, n=3; *p<0.05, **p<0.01, ***p<0.001 compared with TRA-BT solution)
